# Suicidal Attempt among Psychiatry Patients Presented to the Department of Emergency of a Tertiary Care Centre: A Descriptive Cross-sectional Study

**DOI:** 10.31729/jnma.8156

**Published:** 2023-05-31

**Authors:** Prerna Jha, Sandip Subedi, Sreya Paudyal, Prem Prasad Panta

**Affiliations:** 1 Department of Psychiatry, Everest Hospital, New Baneshwor, Kathmandu, Nepal; 2 Department of Psychiatry, Universal College of Medical Sciences and Teaching Hospital, Bhairahawa, Rupandehi, Nepal; 3 Department of Community Medicine, KIST Medical College and Teaching Hospital, Mahalaxmi, Lalitpur, Nepal

**Keywords:** *comorbidity*, *cross-sectional studies*, *prevalence*, *psychosocial factors*, *suicide attempt*

## Abstract

**Introduction:**

More than 700, 000 people die due to suicide every year. Suicide is the fourth leading cause of death among 15-29 year-olds. A total of 77% of global suicides occur in low- and middle-income countries. There is an increasing number of suicide all over the world. There is limited data regarding this issue. The available data are based on police reports or on specific populations. The aim of this study was to find out the prevalence of suicidal attempts among psychiatry patients presented to the Department of Emergency of a tertiary care centre.

**Methods:**

A Descriptive cross-sectional study was done in a tertiary care centre from 11 January 2019 to 11 July 2020 after taking Ethical approval from the same institute. Beck Suicide Intent Scale, MINI-7, IPDE and PLESS were used to assess suicidal intent, psychiatric comorbidities, personality disorder and life stress event scores respectively. Bronfenbrenner's Social Ecological Model was used to access various stressors. Point estimate and 95% Confidence Interval were calculated.

**Results:**

The Prevalence of suicidal attempts among psychiatry patients in the emergency department was 265 (24.50%), (21.66-26.74, 95% Confidence Interval). The majority were females 135 (51%). The majority attempted at home 238 (89.81%). Poisoning was the most common mode of attempting suicide.

**Conclusions:**

The Prevalence of suicidal attempts among psychiatry patients was higher than in the other studies done in similar settings.

## INTRODUCTION

More than 700 000 people die due to suicide every year.^1^ Suicide is the fourth leading cause of death among 15-29 year-olds.^2^ A total of 77% of global suicides occur in low- and middle-income countries. Ingestion of pesticides, hanging and firearms are among the most common methods of suicide globally.^3^ Females seemed to be the most affected, with a 36.8% increase in suspected suicides and attempts by poisoning.^4^

Nepal does not have reliable data related to suicide and attempted suicide. The available data are based on police reports or on specific populations.^5^ There is an increase in the number of suicide all over the world and Nepal is not exceptional. This study is done during the COVID-19 pandemic time, the findings may reflect the pandemic issues related to suicide.

The aim of this study was to find out the prevalence of suicidal attempts among psychiatry patients presented to the Department of Emergency of a tertiary care centre.

## METHODS

A descriptive cross-sectional study was conducted among patients presented to the Department of Emergency, Universal College of Medical Sciences and Teaching Hospital from 11 January 2019 to 11 July 2020. All the patients who presented to the Department of Emergency with suicidal attempts were included in the study. Those with incomplete data and leave against medical advice were excluded from the study. Convenience sampling method was used. The sample size was calculated using the following formula:


$$\begin{array}{ll} \text{n} & ={\text{Z}}^{2}\times \frac{\text{p}\times \text{q}}{{\text{e}}^{\text{2}}}\\ & =1.96^{2}\times \frac{0.50\times 0.50}{0.03^{2}}\\ & =1068 \end{array}$$

Where,

n = minimum required sample size
Z = 1.96 at 95% Confidence Interval (CI)
p = prevalence taken as 50% for maximum sample size calculation
q = 1-p
e = margin of error, 3%

The calculated sample size was 1068. However, a 1095 sample size was taken. The written consent form was taken from the patient. Proforma was used for data collection. Beck's suicide intent scale (SIS),^6^ Mini International Neuropsychiatric Interview (MINI-7),^7^ International Personality Disorder Examination (IPDE-ICD-10 module), resumptive Stressful Life Events Scale (PSLES),^8^ Bronfenbrenner's Social-Ecological Model^9^ was used.

Data were entered in Microsoft Excel 2016 and analysis was done using IBM Statistics SPSS 16.0. Point estimate and 95% CI were calculated.

## RESULTS

Among 1095 patients, the prevalence of suicidal attempts in psychiatry patients was 265 (24.20%), (21.66-26.74, 95% CI). Among them, 81 (30.61%) were unmarried (Table 1).

**Table 1:** Marital Status of the patients (n= 265).

Marital status	n (%)
Married	165 (62.3)
Unmarried	81 (30.61)
Widow	7 (2.63)
Divorced	6 (2.31)
Separated	6 (2.30)

**Figure 1:**
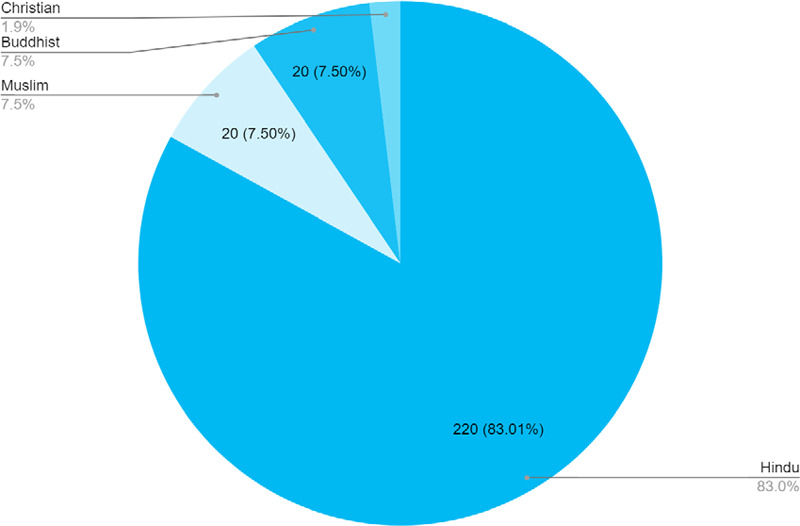
Distribution by religion (n= 265).

In this study, a maximum of 220 (89%) suicide attempters were followers of the Hindu religion, followed by Muslim 20 (7%) (Figure 1).

On the basis of Education status, 94 (35.50%) were illiterate (Table 2).

**Table 2:** Distribution by Education (n= 265).

Education status	
Literate	171 (64.51)
Illiterate	94 (35.50)

In this study, the majority of patients were unemployed which included 84 (31.70%) (Table 3).

**Table 3:** Distribution by occupation (n= 265).

Occupation	n (%)
Unemployed	84 (31.70)
Service	39 (14.74)
Agriculture	34 (12.83)
Housewife	31 (11.70)
Self-employed	28 (10.67)
Student	27 (10.21)
Business	15 (5.71)
Retired	7 (2.60)

In this study, 238 (89.88 %) attempted suicide at home (Table 4).

**Table 4:** Place of suicide attempt (n= 265).

Place of suicide attempt	n (%)
Home	238 (89.88)
Garden	18 (6.81)
Office	3 (1.11)
Hostel	2 (0.82)
Jail	2 (0.88)
Jungle	1 (0.47)
Train Station	1 (0.46)

Using MINI Scale, it was found that 154 (58.11%) of patients had psychiatric comorbidities Major depressive episode current was seen in 64 (24.2%), followed by Alcohol use disorder 27 (10.22%) (Table 5).

**Table 5:** Assessment of psychiatric comorbidities by MINI-7t (n= 265).

MINI	n (%)
Major depressive episode current	64 (24.20)
Alcohol use disorder	27 (10.22)
Major depressive disorder recurrent	23 (8.795)
Generalized anxiety disorder	16 (621)
Schizophrenia current	9 (3.49)
Substance-induced psychotic disorder	5 (1.98)
Major depressive disorder with psychotic feature (current)	3 (1.12)
Bipolar I disorder current	2 (0.85)
Psychotic disorder current and lifetime	1 (0.48)
Antisocial personality disorder	1 (0.48)
Bipolar I disorder with psychotic features (current)	1 (0.12)
Substance use disorder(non-alcohol)	1 (0.48)

## DISCUSSION

In the present study the prevalence of suicide is 20% but according to one of the study, the suicide ideation prevalence varied from 1 to 20% and it varied with study population, geography, age group, gender, and other factors.^10^ In the present study majority of the suicide attempters (53%) were below 30 years of age was similar to the findings of Shakya (60%) and Subba (>60%).^1^ In the present study a female predominance was seen (51.3%). Female predominance was seen in a study conducted by a study.^2^ While some Western and Indian studies showed female predominance, other Western and Indian studies showed male predominance. In another study conducted on 73 patients of intentional self-harm in 2010 found 69.9% were females. In another study among 173 cases of Suicide attempts conducted in Pokhara reported more than two-thirds of the total cases were females.^11^

Out of a total of 477 suicide attempters, 64% were females as stated by Suresh Kumar in his study conducted in Kerala, India in 2001.^4^ Chowdhary studied pesticide poisoning in 5,178 non-fatal Deliberate selfharm cases for 3 years and noted females (63.6%) had predominance over males (36.4%).^12^

In the present study majority of suicide, attempters were from the Nuclear family (66.4%), Joint family, and Extended family. Kulkarni. conducted a case-control study in 100 suicide attempters noted 49% resided in nuclear families, 18% in extended families and 33% in joint families. Some Indian studies have reported the majority of subjects live in nuclear families.^13^

In the present study, 65.5% were literate, 22% had at least passed SLC, 18.9% were secondary, 8.7% had bachelor's degrees and 35.5% were illiterate. This is in accordance with another study which reported that suicide attempts with different methods of poisoning is seems to be more common in a higher level of education whereas, suicide attempts by self-burn and hanging are seen more commonly by illiterate people.^7^ Similarly another study suggested that suicide victims were significantly more often to have a higher education, Persons with higher school attainment, compared with those with a maximum primary school degree, had significantly increased odds ratios of dying from suicide.^14^

In the present study as mentioned in tools, Bronfenbrenner's Social Ecological Model has been used to know the various stressor in suicide attempters. It includes the microsystem, mesosystem, exosystem and individual. The microsystem is any context in which the individual has direct contact and interaction with entities such as the family, the school and peer groups. Examples are Family Conflicts, Large loans, Breakups, the demise of loved ones etc. The mesosystem, on the other hand, refers to the relationships between the microsystems and their influence on each other, parents and romantic partners, while the exosystem refers to those settings not directly involving the individual, but which still exert an impact on him/her example of exosystem includes workplaces and socioeconomic status. The result suggested in this study were Microsystem (45.3%), Macrosystem (20.4%), Individual (20.4%), and Exosystem (13.6%). Similar Results found in a Study conducted in 2012, suggested majority (43%) represented the microsystem of total participants out of which 34% were black participants and 33.6% were coloured participants, resources related to the macrosystem represented 20.2% of the total population and Exosystem represents 1.7%.^5^ Interpersonal difficulties have also been reported as the most common stressor for attempting suicide by various Indian and foreign studies.^15^

The limitation of this study is, it is a single-centred study, and the findings cannot be generalized to the whole population. Further studies can be done to justify the associating factors.

## CONCLUSIONS

The prevalence of suicidal attempters was higher than in the other studies done in similar settings. Psychiatric disorders were significantly present in patients with suicidal attempts. The findings of this study are likely to have a notable impact on the field of psychiatry. In Nepal very few studies have addressed the issue of comorbidity, psychosocial factors and demographic details, hence future prospective studies with a large sample size are needed to address the issue of comorbidity. The Nepali population is highly diverse in terms of socio-cultural background. Considering these aspects it is fair to say that there is a great need for well-designed, cross-sectional studies regarding attempted suicide in Nepal.

## References

[1] World Health Organization. (2014). Preventing suicide: a global imperative [Internet].

[2] Katuwal N, Shrestha DB, Adhikari SP, Oli PR, Budhathoki P, Amatya R (2021). Study on prevalence of suicidal ideation and risk factors of suicide among patients visiting psychiatric OPD at Shree Birendra Hospital, Kathmandu Nepal.. PLoS One.

[3] Ruiz P, Sadock VA (2009). Kaplan and Sadock's Comprehensive Textbook of Psychiatry..

[4] Hendin H, Vijayuakumar L, Bertolote JM, Wang H, Phillips MR, Pirkis J, Hendin H, Phillips MR, Vijayakumar L, Pirkis J, Wang H, Yip P (2008). Suicide and suicide prevention in Asia..

[5] Thapaliya S, Sharma P, Upadhyaya K (2018). Suicide and self-harm in Nepal: a scoping review.. Asian J Psychiatr..

[6] Sheehan DV, Lecrubier Y, Sheehan KH, Amorim P, Janavs J, Weiller E (1998). The Mini-International Neuropsychiatric Interview (M.I.N.I.): the development and validation of a structured diagnostic psychiatric interview for DSM-IV and ICD-10.. J Clin Psychiatry..

[7] Bastianon CD, Klein EM, Tibubos AN, Brahler E, Beutel ME, Petrowski K (2020). Perceived stress scale (PSS-10) psychometric properties in migrants and native Germans.. BMC Psychiatry..

[8] Singh G, Kaur D, Kaur H (1984). Presumptive stressful life events scale (psles) - a new stressful life events scale for use in India.. Indian J Psychiatry..

[9] Thapaliya S, Gupta AK, Tiwari S, Belbase M, Paudyal S (2018). Pattern of suicide attempts in Southern Nepal: a multi-centered retrospective study.. Med Phoenix..

[10] Marahatta K, Samuel R, Sharma P, Dixit L, Shrestha BR (2017). Suicide burden and prevention in Nepal: the need for a national strategy.. WHO South-East Asia J Public Health..

[11] Blackmore ER, Munce S, Weller I, Zagorski B, Stansfeld SA, Stewart DE (2008). Psychosocial and clinical correlates of suicidal acts: results from a national population survey.. Br J Psychiatry..

[12] Alves V de M, Francisco LCF de L, Belo FMP, de Melo Neto VL, Barros VG, Nardi AE (2016). Evaluation of the quality of life and risk of suicide.. Clinics..

[13] World Health Organization. (1993). The ICD-10 classification of mental and behavioral disorder diagnostic criteria for research [Internet]..

[14] Choo CC, Harris KM, Chew PKH, Ho RC (2019). Clinical assessment of suicide risk and suicide attempters' self-reported suicide intent: a cross-sectional study.. PLoS One.

[15] Bi B, Liu W, Zhou D, Fu X, Qin X, Wu J (2017). Personality traits and suicide attempts with and without psychiatric disorders: analysis of impulsivity and neuroticism.. BMC Psychiatry..

